# Bullying in Orthopaedic Surgery: A Survey of US Orthopaedic Trainees and Attending Surgeons

**DOI:** 10.5435/JAAOSGlobal-D-23-00007

**Published:** 2023-05-02

**Authors:** Monica M. DiFiori, Sanchita S. Gupta, Lisa K. Cannada, Kevin Y. Pei, Michaela A. Stamm, Mary K. Mulcahey

**Affiliations:** From the Department of Orthopaedic Surgery and Sports Medicine, Temple University Hospital, Philadelphia, PA, (Dr. DiFiori); Tulane University School of Medicine, New Orleans, LA (Ms. Gupta); Department of Orthopaedics, University of North Carolina and Novant Health Orthopaedic Fracture Clinic, Hughston Clinic, Jacksonville, FL (Dr. Cannada); Graduate Medical Education, Parkview Health, Fort Wayne, Indiana (Dr. Pei); Department of Orthopaedic Surgery, Tulane University School of Medicine, New Orleans, LA (Dr. Stamm and Dr. Mulcahey).

## Abstract

Bullying is a notable problem in surgery, creating a hostile environment for surgeons and trainees, and may negatively affect patient care. However, specific details regarding bullying in orthopaedic surgery are lacking. The primary aim of this study was to determine the prevalence and nature of bullying within orthopaedic surgery in the United States.

**Methods:**

A deidentified survey was developed using the survey created by the Royal College of Australasian Surgeons and the validated Negative Acts Questionnaire-Revised survey tool. This survey was distributed to orthopaedic trainees and attending surgeons in April 2021.

**Results:**

Of the 105 survey respondents, 60 (60.6%) were trainees and 39 (39.4%) were attending surgeons. Although 21 respondents (24.7%) stated they had been bullied, 16 victims (28.1%) did not seek to address this behavior. Perpetrators of bullying were most commonly male (49/71, 67.2%) and the victims' superior (36/82, 43.9%). Five bullying victims (8.8%) reported the behavior, despite 46 respondents (92.0%) stating that their institution has a specific policy against bullying.

**Conclusion:**

Bullying behavior occurs in orthopaedic surgery, with perpetrators being most commonly male and the victims' superiors. Despite the fact that an overwhelming majority of institutions have policies against bullying, the reporting of such behavior is lacking.

The presence of bullying in surgery has been well documented within the United States^1-3^ and internationally.^4,5^ In a survey completed by surgery trainees across all surgical specialties in the United Kingdom and the Republic of Ireland, approximately 48% of respondents described the surgical workplace as an environment with a moderate, high, or very high degree of bullying behavior.^4^ Bullying in the workplace has been associated with poor mental health.^6-8^ Specifically, two independent studies in Germany and Denmark found that bullying in the workplace was a risk factor of depression.^7,8^

In an online, anonymous survey of 582 US surgical residents across all surgical subspecialties, 57% of respondents either screened positive for post-traumatic stress disorder or were at risk of developing post-traumatic stress disorder.^9^ The most frequently cited stressor was bullying by attendings, fellow residents, nurses, and other staff members. The authors further argued that bullying is related to physician burnout.^9^ A survey of US urology residents demonstrated that 90% of respondents experienced bullying; 15% stated bullying affected their performance; and 36% stated it affected patient care.^2^ Based on these findings, bullying within surgical training is clearly a notable issue, the effects of which warrant additional investigation.

Discrimination, bullying, and harassment are a concern within the field of orthopaedic surgery, as detailed by a survey of American Academy of Orthopaedic Surgeons (AAOS) members conducted by Samora et al.^3^ Survey respondents included trainees and attending surgeons. Most notably, 66% of respondents had experienced discrimination, bullying, and/or harassment; 55% specifically reported experiencing bullying. After reporting the behavior, 43% of respondents stated the behavior continued and 20% stated they were further victimized.^3^

In addition, bullying may deter medical students (especially women and students underrepresented in medicine) from pursuing a career in orthopaedic surgery, which may contribute to the overall lack of diversity in the field.^10^ Orthopaedic surgery currently has the lowest percentage of female residents (15.4%)^11^ of all medical specialties, and female orthopaedic surgeons comprise only 5.8% of AAOS society membership.^12^ Moreover, non-White practicing orthopaedic surgeons account for only 14.1% of AAOS society membership.^12^ Bullying contributes to a toxic atmosphere, which is a barrier of entry for women and underrepresented minorities. At a time when orthopaedic surgery is the least gender-diverse specialty in medicine,^11^ it is paramount to explore all avenues to increase the diversity of the field.

With a better understanding of how bullying plays a role in the culture of orthopaedic surgery, this behavior can be addressed in a direct and efficient manner. More information is needed to define the extent of the problem and provide methods to decrease the occurrence of bullying in orthopaedics. The purpose of this study was to determine the prevalence and nature of bullying within orthopaedic surgery among residency programs in the United Sates to further delineate the problem and provide suggestions to improve the culture of orthopaedic surgery.

## Methods

This study was approved by the Institutional Review Board at the senior author's institution. For the purposes of this study, bullying was defined as a situation where one or several individuals persistently over a period of time perceive themselves to be on the receiving end of negative actions from one or several persons, in a situation where the target of bullying has difficulty in defending him or herself against these actions. This definition is included as part of the validated Negative Acts Questionnaire-Revised (NAQ-R) survey tool, which was provided to all subjects who participated in this study.^13^

A deidentified, anonymous online survey was developed using the qualitative survey created by the Royal College of Australasian Surgeons^5^ and the NAQ-R survey tool,^13^ with additional demographic questions. The NAQ-R survey tool uses a raw score to categorize respondents into three groups: 20 to 32 points correspond to someone who is “not bullied or rarely bullied,” 33 to 44 points correspond to someone who is “sometimes bullied,” and greater than 45 corresponds with “a victim of workplace bullying.”

Using Qualtrics (Seattle), the survey was distributed via e-mail to nine program directors who are also members of the Collaborative Orthopaedic Educational Research Group. Program directors were then asked to distribute the survey to their residents, fellows, and other attending surgeons at their respective programs, totaling 251 potential survey respondents. Responses were not required for each question in the survey. Follow-up e-mails were sent to program directors at two and 4 weeks after the initial e-mail to encourage participation. Residents, fellows, and faculty were able to complete the survey between April 5, 2021, and May 24, 2021. Descriptive statistics were used to evaluate all survey question responses. Further data analyses were performed using chi square analysis, Spearman and Pearson correlation analysis, Bartlett test, Bonferroni test, two-sample *t*-test, and Student *t*-test.

## Results

### Survey Participant Demographics

One hundred five of 251 surgeons (41.8%) responded, of which 58 (58.6%) were residents, 39 (39.4%) were attending surgeons, and 2 (2.0%) were fellows. A majority of survey respondents (56/83, 67.4%) were between the ages of 26 and 35 years. Sixty-nine respondents (84.2%) identified as male and nine (11.0%) as female. Sixty-two of 85 respondents (73.0%) identified as Caucasion or White. Two of 82 respondents (2.4%) identified as of Hispanic, Latinx, or Spanish origin. Sixty-four of 84 respondents’ programs (76.2%) were located in the Northeast. Fifty respondents (60.2%) were married, 24 (28.9%) were single, and nine (10.9%) were partnered (Table 1). Participants who stated they were partnered were more likely to have a higher NAQ-R raw score compared with those were not partnered (*P* = 0.005).

**Table 1:** Demographics of all Survey Respondents

Age (n = 83)	
26-30 yr	34.94% (29)
31-35 yr	32.53% (27)
36-40 yr	10.84% (9)
41-45 yr	8.43% (7)
46-50 yr	4.82% (4)
51-55 yr	3.61% (3)
56-60 yr	3.61% (3)
61-65 yr	1.20% (1)
Sex (n = 82)	
Male	84.15% (69)
Female	11.98% (9)
Transgender Female	1.22% (1)
Transgender Male	0.0% (0)
Sex nonconforming/Nonbinary	1.22% (1)
Decline to answer	2.44% (2)
Race (n = 85)	
Caucasian or White	72.94% (62)
Asian	14.12% (12)
Black or African American	3.53% (3)
Native Hawaiian or Pacific Islander	1.18% (1)
American Indian or Alaskan Native	1.18% (1)
Ethnicity (n = 82)	
Hispanic, Latinx, or Spanish origin	2.44% (2)
Not of Hispanic, Latinx, or Spanish origin	87.80% (72)
Prefer not to say	9.76% (8)
Geographic location (n = 84)	
Northeast (PA, NY, CT, MA, NH, VT, ME, RI, or NJ)	76.19% (64)
South (TX, AL, MS, LA, OK, AR, GA, SC, NC, FL, TN, KY, VA, WV, MD, Washington, DC, or DE)	15.48% (13)
West (CA, WA, OR, NV, AZ, UT, CO, NM, WY, ID, or MT)	7.14% (6)
Pacific (HI or AK)	1.19% (1)
Midwest (ND, SD, NE, KS, MO, IA, MN, WI, MI, IL, IN, or OH)	0.0% (0)
Relationship Status (n = 83)	
Married	60.24% (50)
Single	28.92% (24)
Partnered	10.84% (9)
Widowed/Widower	0.0% (0)
Divorced	0.0% (0)
Average hours worked per week (n = 96)	
<40 hr/wk	2.08% (2)
40-60 hr/wk	23.96% (23)
61-70 hr/wk	23.96% (23)
71-80 hr/wk	30.21% (29)
81-90 hr/week	9.38% (9)
91-100 hr/wk	8.33% (8)
101-110 hr/wk	2.08% (2)
>110 hours/wk	0.0% (0)

**Figure 1:**
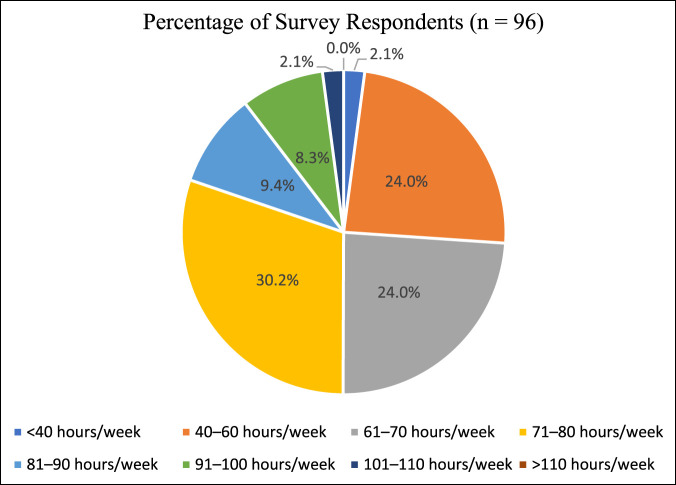
Average hours worked per week of all survey participants.

Over the 6 months prior to completing the survey, 29 respondents (30.2%) worked an average of 71 to 80 hours per week. Nineteen respondents (19.8%) worked over 80 hours per week (Figure 1). A higher average hours worked per week was associated with a higher NAQ-R raw score (*P* = 0.0057).

Thirty-three of the residents and fellows (55.0%) who responded to the survey identified their program setting as university or academic while 27 (45.0%) identified their program setting as a community hospital. Twenty-six residents (44.1%) were in their first or second postgraduate year of clinical training, 10 (17.0%) were in their third postgraduate year, and 21 (35.6%) were in their fourth or fifth postgraduate year. Two (3.4%) were in their sixth postgraduate year or higher (Table 2).

**Table 2:** Resident and Fellow Program Type and Postgraduate Year

Program type	
University or academic	55.0% (33/60)
Community hospital	45.0% (27/60)
PGY	
PGY1 and PGY2	44.1% (26/59)
PGY3	17.0% (10/59)
PGY4 and PGY5	35.6% (21/59)
≥PGY6	3.4% (2/59)

PGY = Postgraduate year

According to the NAQ-R, 10 survey respondents (12.0%) were the victim of workplace bullying and 14 (16.9%) were sometimes bullied (Table 3). Demographic information including age, sex, geographic region, stage of training, race, and ethnicity did not have a statistically significant association with self-reports of being a victim of bullying (Table 4).

**Table 3:** NAQ-R Scores

Proportion of Survey Participants	NAQ-R Raw Score	NAQ-R Classification
71.1% (59/79)	20-33 points	Not bullied or rarely bullied
16.9% (14/79)	33-44 points	Sometimes bullied
12.1% (10/79)	≥45 points	Victim of workplace bullying

NAQ-R = Negative Acts Questionnaire-Revised

**Table 4:** Pearson Chi Square Analysis of the Relationship Between Survey Respondents' Demographic Information and Self-Reporting of Bullying Victimization

Demographic Variable	*P*-value
Age	0.837
Sex	0.167
Geographic region	0.088
Stage of training	0.930
Race	0.871
Ethnicity	0.282

Among attendings, 27 (75.0%) identified their practice setting as university, 5 (13.9%) as university-affiliated, 3 (8.3%) as hospital-employed, and one (2.8%) as private practice. Thirteen attendings (36.1%) who responded to the survey were in practice 6 years or less (Table 5). The number of years in practice as an attending was inversely proportional to their NAQ-R raw score (*P* = 0.0246). Attendings in practice between 6 and 10 years were more likely to have a higher mean score on the NAQ-R in comparison with attendings in practice for less than 6 years or greater than 10 years (*P* = 0.035).

**Table 5:** Attending Surgeon Career Demographics

Practice setting (n = 36)	
University	75.0% (27)
Private practice	2.8% (1)
University-affiliated	13.9% (5)
Hospital-employed	8.3% (3)
Years in practice (n = 36)	
0-5	36.1% (13)
6-10	22.2% (8)
11-20	27.8% (10)
21-30	13.9% (5)
>30	0.0% (0)
Academic rank (n = 36)	
Professor	25.0% (9)
Associate Professor	25.0% (9)
Assistant Professor	50.0% (18)
Administrative positions (n = 34)	
Department Chair	8.8% (3)
Associate/Assistant/Vice chair	8.8% (3)
Program Director	14.7% (5)
Associate/Assistant Program Director	8.8% (3)
Section/Division Chief	14.7% (5)
Dean	0.0% (0)
Associate/Assistant/Vice Dean	0.0% (0)
Clerkship Director	5.9% (2)
Associate Clerkship Director	0.0% (0)
Operating Room Committee Member	8.8% (3)
Hospital System Committee Position	29.4% (10)

Eighteen of the 36 attendings (50.0%) who responded to the survey were assistant professors, 9 (25.0%) were associate professors, and 9 (25.0%) were professors. Thirty-four attendings (87.2%) held an administrative position, of whom 10 (29.4%) held a hospital system committee position, 5 (14.7%) were section or division chiefs, and 5 (14.7%) were program directors (Table 5).

### Bullying Behavior and Reporting

Two respondents (2.4%) stated they were bullied several times a week, 4 (4.7%) were bullied “now and then,” 15 (17.7%) were bullied only rarely at work, and 64 (75.3%) had not been bullied at work. Of those who stated they were bullied, the perpetrators were more frequently male (19/26, 73.1%) and the victims' superior (19/34, 55.9%). An additional 10 respondents (29.4%) were bullied by their colleagues. Three respondents (8.8%) were bullied by nursing staff, and two respondents (5.9%) were bullied by patients. Respondents who stated they have been bullied in the past six months were more likely to have a higher average NAQ-R raw score (*P* < 0.001).

**Figure 2:**
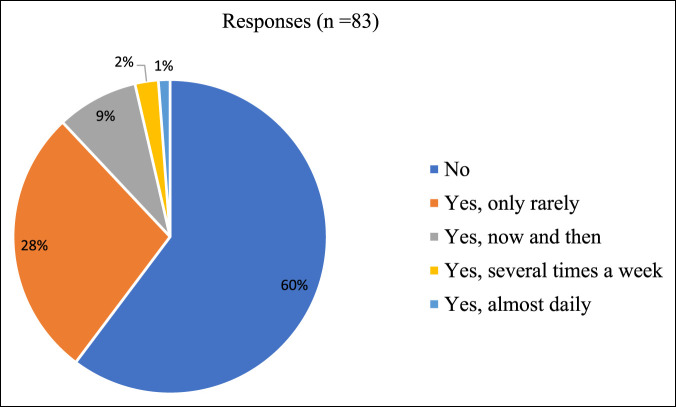
Survey responses: Have you witnessed a colleague being bullied in the past 6 months?

Thirty-three of 83 respondents (39.8%) stated they had witnessed a colleague being bullied at work over the past 6 months while 50 (60.2%) had not (Figure 2). Of the respondents who stated they have witnessed a colleague being bullied at work, the perpetrator was most commonly identified as a colleague (21/48, 43.8%). Nine perpetrators (18.8%) were identified as an immediate superior, and 8 perpetrators (16.7%) were identified as other superiors/managers (ie, program director, chair). When asked to describe the sex of their colleagues' perpetrators, 30 (66.7%) were male while 15 (33.3%) were female. Respondents who stated they have witnessed a colleague being bullied at work were also more likely to have a higher NAQ-R raw score (*P* = 0.004).

**Figure 3:**
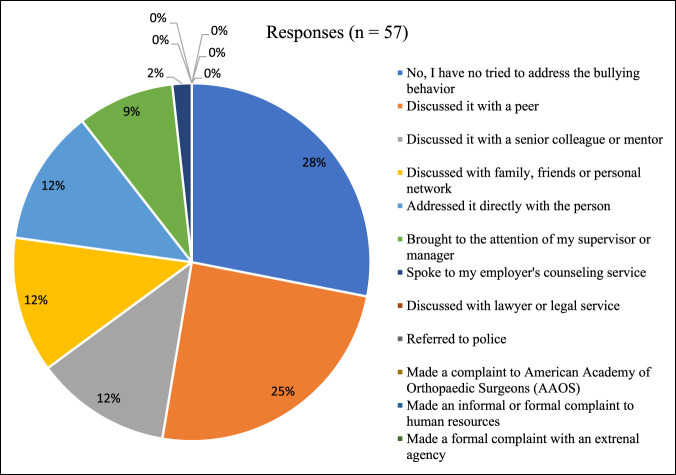
Response to bullying behavior

Sixteen of 57 respondents (28.1%) stated they had not tried to address the bullying behavior. Fourteen respondents (24.6%) discussed it with a peer. No one made an informal or formal report to human resources, or discussed it with a lawyer or legal service (Figure 3). When asked about the result of the action(s) they took, 8 respondents (34.8%) stated that the behavior stopped while 7 (30.4%) stated that the behavior continued. The most common potential barrier to taking action against bullying behavior identified by respondents was the effect on their future career options (14/61, 23.0%). The second most common barrier was the loss of reputation for self (13/61, 21.3%) (Table 6).

**Table 6:** Addressing Bullying Behavior: Barriers and Outcomes Within the United Sates Orthopaedic Surgery Residency Programs.

Barriers to addressing bullying behavior (n = 61)	
Effect on future career options	23.0% (14)
Loss of reputation of self	21.3% (13)
Loss of support of supervisors, colleagues, friends, partner, and family	13.1% (8)
The stress associated with filing a complaint and enduring an investigation	13.1% (8)
Fear of being blamed	8.2% (5)
Potential for victimization	8.2% (5)
Concern of not being believed or taken seriously by management	6.6% (4)
Loss of reputation of perpetrator	6.6% (4)
Results of actions taken against bullying (n = 23)	
This behavior stopped	34.8% (8)
This behavior continued	30.4% (7)
Complaint was not pursued by the receiving body	13.0% (3)
I received an apology	8.7% (2)
My employer made changes to the workplace to prevent this behavior in the future	4.4% (1)
There was retaliation for making a complaint	4.4% (1)
I left my job	4.4% (1)
I received compensation	0.0% (0)
Complaint has not yet been finalized	0.0% (0)

### Workplace and Bullying

**Figure 4:**
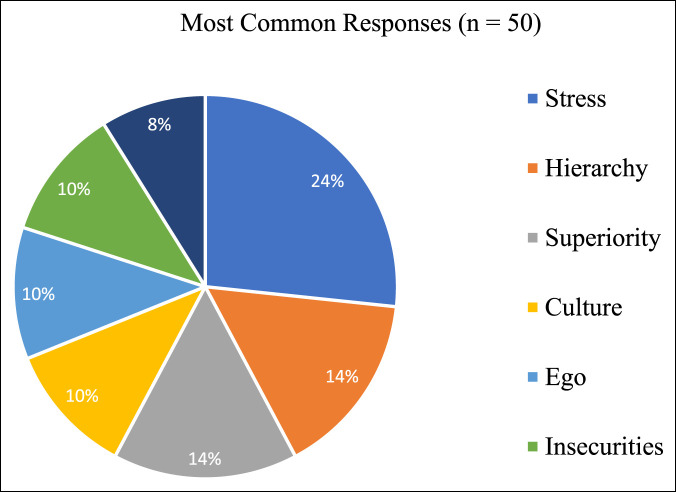
Survey respondents' opinion: Why does bullying occur in the workplace?

Four attendings (20.0%) with an administrative position stated they had received a report about bullying behavior in the past 2 years, while 16 (80.0%) stated they had not received a report about bullying behavior during the same time period. Eleven attendings (55.0%) stated they have a zero-tolerance policy for bullying behavior, recommending disciplinary action after the first substantiated report. Forty-six survey participants (92.0%) stated there was a policy in place to specifically address bullying behavior within their institution, whereas 4 respondents (8.0%) stated there was no policy in place at their institution. Survey respondents were also asked, “Why do you think bullying occurs amongst surgeons in the workplace?” The most common responses included “stress” (24.0%), “hierarchy” (14.0%), and “superiority” (14.0%). Four respondents (8.0%) stated bullying does not occur (Figure 4).

## Discussion

The results of this survey demonstrate that bullying behavior occurs in orthopaedic surgery departments across the United Sates. Victims and witnesses of bullying most commonly identified the perpetrators as male and the victims' superiors. No statistical association was observed between victims of bullying and their sex, race, ethnicity, geographic location, and age. Attendings in practice between 6 and 10 years were more likely to be a victim of bullying compared with their peers. Despite the fact that an overwhelming majority of institutions have policies in place against bullying, the reporting of such behavior is lacking. The concern for future career options and the victims' reputation were the two most commonly cited barriers to reporting the behavior.

This study sample is reflective of the AAOS society membership, as 84.2% of survey respondents identify as male, and 92.2% of AAOS members are male. Approximately three quarters of survey respondents identified as Caucasian or White, which is slightly lower than the 85.9% of AAOS members who identify as Caucasian or White.^12^ In a sample that is largely male and White, it is difficult to delineate how bullying behavior may impact the field of orthopaedics on a detailed level, especially those under-represented in this field. Although 24.7% of survey participants in this study reported experiencing bullying behavior, this represents progress within the field, as a previous survey study found 55% of AAOS members experience bullying.^3^ It is important to note that this study conducted by Samora et al. specifically surveyed women and underrepresented minorities within AAOS.

This study did not find a statistically significant association between respondents' age, sex, race, or ethnicity, and NAQ-R scores or identifying as a victim of bullying. However, previous literature has demonstrated that female surgeons were more likely than their male colleagues to experience bullying.^1^ Furthermore, in a similar study of 6956 general surgery residents using the Short-Negative Act Questionnaire, women were more likely to be the victims of bullying as compared to men. Additionally, residents who identified as a racial and/or ethnic minority were more likely to be the victim of bullying behavior.^14^ While our study differs from the previous literature, it is possible that our homogenous sample prohibits a true examination of the statistical associations between NAQ-R scores and the demographic characteristics of bullying victims.

The impact of bullying is well established. In a recent study by Hu et al,^15^ general surgery residents who experienced “mistreatment” were statistically more likely to experience burnout and suicidal thoughts. The authors also found a stepwise relationship between the frequency of mistreatment and increased suicidal thoughts. Zhang et al^14^ also demonstrated that general surgery residents who were frequently bullied had increased suicidal thoughts and burnout when compared with their peers who were not frequently bullied. Bullying of residents can also cause medical errors, patient harm, and undue financial costs to the healthcare system, which can include replacement of individuals who leave their positions secondary to bullying, or from the consequences of medical errors and subsequent litigation.^16^ In our study, one respondent (4.35%) who experienced bullying in the workplace left their position as a result.

Respondents to this survey most commonly identified the perpetrators of bullying as male and the victim's superior. These results are similar to a survey conducted by Best Practice Australia, which found trainees and attending surgeons with less than 10 years of experience were subjected to higher rates of bullying (54% and 45%, respectively). Meanwhile, only 31% of bullying victims were attending surgeons with greater than 10 years of experience. The perpetrators of bullying were most commonly identified as male in this study as well.^5^ Zhang et al^14^ found that attending surgeons were the most common perpetrators of bullying behavior in general surgery. Interestingly, the number of years in practice as an attending was found to be inversely proportional to NAQ-R scores in the study presented here. Meaning, junior attendings were more likely to be the victims of bullying when compared with more senior attendings. Attendings who are younger in their practice may seem less competent because of lack of experience, making them a target by their more senior colleagues. As Pei and Cochran^17^ described, junior faculty and trainees are most at risk to experience bullying behavior due to the power imbalance embedded in surgical training and academic advancement—career opportunities for those lower in the surgical hierarchy are controlled by a select few at the top.

Ninety-two percent of survey participants stated there was a policy which specifically addresses bullying behavior within their institution; however, less than 9% of victims reported the behavior to their supervisor. In a systematic review of 25 survey studies regarding discrimination, bullying, and sexual harassment within surgery in the United States and abroad, Gianakos et al^18^ identified the underreporting of such behavior as a major hurdle to curbing bullying in the workplace. Using the NAQ-R to assess bullying among general surgery residents and attendings, similar trends were found: 30.0% of residents and 20.0% of attendings did not address bullying behavior. Only 28.3% of residents brought the behavior to the attention of their supervisor, as compared with 44.0% of attendings.^1^ The underreporting of bullying behavior in orthopaedic surgery and other surgical specialties is not only concerning, but most importantly, it is impeding the eradication of bullying behavior from the culture of surgery.

Approximately a quarter of this survey's respondents attributed stress to the bullying that occurs in orthopaedics, and another 14.0% of respondents referenced hierarchy. When general surgery residents and attendings were asked the same question, stressful work and strict hierarchy were among the top three answers.^1^ The hierarchical nature of orthopaedic surgery lends itself to the perpetuation of bullying because it deters victims from reporting this behavior. As demonstrated in our study and previous literature, victims are often positioned lower within the hierarchy of surgery, and thus less comfortable coming forward.^5,14,18^

The most common potential barriers to taking action against bullying behavior identified by respondents in this survey were the effect on their future career options and damage to the victim's reputation. In Pei's National Assessment of Workplace Bullying in Among Academic Surgeons in the Unites States, residents and attendings also reported the effect of future career options and damage to the victim's reputation as the two most common barriers to reporting bullying behavior.^1^ The aforementioned survey conducted by Best Practice Australia demonstrated similar findings: Approximately 45% of victims did not take action against bullying behavior, citing fear of negative effects on their career as the most common reason.^5^ There have been several calls for changes to the reporting processes for bullying, and discrimination and sexual harassment as well.^5,14,16^ For example, improving reporting processes to ensure confidentiality and protect victims against retaliation have been suggested.^5,16,18^ The findings of this study bolster these statements; bullying behavior will continue to detract from the culture of orthopaedics if changes to the reporting process are not made. Additional solutions have been put forth in the literature including the strict implementation of a zero-tolerance policy, the use of an ombudsperson, and training on implicit bias and bystander intervention.^5,10,16,18^

There are several limitations to this study. Given the low response rate (41.8%), it is difficult to make stratified comparisons and compare the results presented here to the current literature. A majority of respondents identified as male; however, previous literature has demonstrated that women are more often the victims of bullying behavior. Thus, while this survey certainly represents the demographics of orthopaedics as a whole, it may not accurately capture rates of bullying behavior in orthopaedic surgery specific to sex, race, ethnicity, nor age. The survey respondents included in this study are associated with nine ACGME-accredited programs, with a majority of those located in the Northeast. Future studies should make a concerted effort to include orthopaedic surgeons and orthopaedic surgeons in training in all geographic regions. This survey is also subject to response bias, as those who experienced bullying may be more likely to respond to the survey. Finally, the survey asked participants to assess bullying experiences over a period of six months, which could lead to recall bias.

## Conclusion

Bullying behavior occurs in orthopaedic surgery departments across the United Sates. Victims and witnesses of bullying identified the perpetrators most commonly as male and the victims' superiors. Despite the fact that an overwhelming majority of institutions have policies in place against bullying, the reporting of such behavior is lacking. The hierarchical nature of orthopaedic surgery lends itself to the perpetuation of bullying because it deters victims and bystanders from speaking up regarding this behavior.
